# 

**Published:** 1897-05-01

**Authors:** 


### EARLY EDITION.

### NOTICE TO CORRESPONDENTS.

All MS., Letters, Books for Review, and other matters intended for the Editor should be addressed The Editor, "The Hospital," The Hospital Building, 28 & 29, Southampton Street, Strand, W.C.
The Editor cannot undertake to return rejected MS., even when accompanied by stamped directed envelope.

**Notice to Advertisers and Subscribers.**

All Advertisements, Orders for Copies of The Hospital, and Business Communications should be addressed to **THE MANAGER, (Not to The Editor),** The Hospital, The Hospital Building, 28 & 29, Southampton Street, Strand, London, W.C.
The Cost of Subscription, post-free, is as follows:—Per week, 2½d.; per quarter, 2s. 8d.; per half-year, 5s. 3d.; per annum, 10s. 6d. Foreign:—Per annum, 15s. 2d.; payable, in all cases, in advance.

**NOTICE.—Vol. XXI. of "The Hospital" (October, 1896, to March, 1897), in handsome cloth binding, is on sale at the Office of this Journal, price 7s. 6d. Cases for binding the Numbers contained in this Volume, 1s. 6d. Index to Vol. XXI., 2½d., post free.**
**The Monthly Part for May will be ready on May 1st. Price 6d., by post 8d.**

### BOOKS RECEIVED.

Scientific Press.

"The Matron's Course: An Introduction to Hospital and Private Nursing." By Miss S. E. Orme.

P. S. King and Son.

"Poor Law Schools: A Criticism of the Report of the Departmental Committee."

H. K. Lewis.

"Compressed Air Illness." By E. Hugh Snell, M.D.

William Clowes and Sons.

"Sanitation and Health." By Brig.-General R. C. Hart, V.C., C.B., revised by Brig.-Surgeon Lieut.-Colonel T. H. Hendley, C.I.E.

Knowledge Office.

Society for the Protection of Birds. Educational Leaflets, part 1.

Smith, Elder, and Co.

"Digestion and Diet." By Sir William Roberts, M.D., F.R.S.

J. and A. Churchill.

"Antiseptic Principles for Nurses." By C. E. Richmond, F.R.C.S.

The Chiswick Press.

"A Protest Against the Modern Development of Unmusical Tone." By Thomas C. Lewis.

"London" Offices.

"The Municipal Year Book for 1897." By Robert Donald.

Philosophical Society of Glasgow.

"Presidential Address on the Pollution of Scottish Rivers." By John Glaister, M.D.

Watts and Co.

"A Plea for the Unborn." By Henry Smith.

**Periodicals Received.**—Westminster Review, Nursing Notes, Happy Home, The Literary Digest, The Surveyor, The Humanitarian, The Sun, The Medical Record (New York), The Charities Review, The Gnyoscope, The Woman's Signal, Guy's Medical Gazette, Industries and Iron, Electrical Review.

### The Student of Medicine.

**APPOINTMENTS.**

J. Grant Andrew, M.B., C.M.Glas., appointed Assistant Surgeon to the Victoria Infirmary, Glasgow. Norman G. Bennett, B.A., B C.Cantab., L.D.S.Eng., appointed Assistant Dental Surgeon to the Dental Hospital of London. Alban Butler, L.R.C.P., L.R.C.S.I., appointed Medical Officer of Health for the Thedwastre Rural District. K. C. Chetwood-Aiken, M.B., M.S.Aberd., appointed Senior House Surgeon to the Royal London Ophthalmic Hospital, Moorfields. James S. Collier, M.D.Lond., B Sc., M.R.C.P., appointed Junior House Physician to the National Hospital for the Paralysed and Epileptic, Queen Square. J. Douglas Cree, M.R.C.S., M.R.C.P., appointed Clinical Assistant to Out-patients at the Chelsea Hospital for Women, Fulham Road. George De Clive-Lowe, L.R.C.P., and S.E., L.F.P.S.G., appointed Medical Officer of Health for Hokiangu, New Zealand. Joseph Francis Duffy, L.R.C.P., L.R.C.S.I., appointed Medical Officer to the Ballinasloe Workhouse. Stephen G. Floyd, M.D., Lond., M.R.C.S.Eng., L.R.C.P., appointed Medical Officer of Health to the Llandrindod Wells Urban District Council. G. M. Fox, L.R.C.P.Lond., M.R C.S.-Eng., appointed Medical Officer to the Workhouse of the Walsall Union. Dr. Gower, appointed Medical Officer for the Stockbridge District of the Wortley Union. George A. Hamerton, M.D., F.R C.S.Eng., appointed Medical Officer to the Inland Revenue Department, Somerset House. H. Cecil Harper, M.R.C.S.Eng., L.R.C.P.Lond., appointed Medical Officer of Health to the Stowmarket Urban District. John S. Holden, M.D., L R.C.S., appointed Medical Officer of Health to the Glemsford Urban District Council. T. W. Jenkins, M.A., M.D.Glasg., appointed Assistant Surgeon to the Victoria Infirmary, Glasgow. A. G. Kewley, B.A.Oxon., L.R.C.S., L.R.C P.Edin., L F.P.S.Glasg., appointed House Surgeon to the Royal Southern Hospital Liverpool. T. R. Kingdon, B.A.Camb., M.R.C.S., L.R.C.P., appointed House Surgeon to Weston-super-Mare Hospital and Dispensary. G. R. Livingston, M.B., C.M., appointed Resident Medical Officer to the Royal Edinburgh Hospital for Sick Children. J. Lockwood, M.R.C.S.Eng., L.S.A., appointed Medical Officer of Health for the Kirkburton Urban District. C. R. Lucas, M.R.C.S.Eng., L.R.C.P.Lond., appointed Medical Officer to the Fairfield House Workhouse of the Chelsea Parish. W. T. McCombe, L.R.C.P., L.R.C.S.Edin., appointed Medical Officer to the Workhouse and the Beccles District of the Wangford Union. Dr. McLaren, appointed Medical Officer for the No. 3 District of the Wortley Union. N. Magoris, M.B., B.S., appointed Clinical Assistant to Out-patients at the Chelsea Hospital for Women, Fulham Road. M. P. Nicholl, M.R.C.S , L R.C.P.Lond., appointed Second Assistant Medical Officer to the St. Marylebone Infirmary, North Kensington. J. E. Parker, M.R.C.S.Eng., L.R.C.P.I., appointed Medical Officer for the Ince-in MakerfieldDistrict of the Wigan Union. Dr. Parkhurst, appointed Medical Officer for the Gedney and Sutton District of the Holbeach Union. E. G. D. Pineo, M.R.C.S., L.R.C.P., appointed Provident Medical Officer to Weston-super-Mare Hospital and Dispensary. Thomas Redmayne, M.B., M.A.Cantab., F.R.C.S., appointed Honorary Assistant Surgeon to the Hastings, St. Leonard's, and East Sussex Hospital. William T. Ritchie, M.B , C.M., appointed Resident Medical Officer to the Royal Edinburgh Hospital for Sick Children. Sidney John Roberts, L.R.C.P.Lond., M.R.C.S.-Eng., appointed Medical Officer and Public Vaccinator to the No. 3 District of the Aston Union. Purves Stewart, M.A., M.B.Edin., M.R.C.S.E., appointed Senior House Physician to the National Hospital for the Paralysed and Epileptic, Queen Square. James A. W. Watts, M.B., B.S.Durh., M.R.C.S.Eng., L.R.C.P.Lond., appointed Medical Officer to the Post Office at Hyde. Meredith Young, M.B., C.M.Edin., D.P.H.Vict., appointed Medical Officer of Health to the Borough of Crewe.

London: The Scientific Press Ltd., 28 & 29, Southampton Street, Strand, W.C.

### STANDARD TEXT-BOOKS FOR NURSES.

12mo., Illustrated with 73 Engravings in the Text, and 9 coloured and half-tone Plates. Price 7s. 6d. net.

**PRACTICAL POINTS IN NURSING.** For Nurses in Private Practice. With an Appendix containing Rules for Feeding the Sick; Recipes for Invalid Foods and Beverages; Weights and Measures; Dose List; and a full Glossary of Medical Terms and Nursing Treatment. By EMILY A. M. STONEY, Superintendent of Training School for Nurses, Carney Hospital, South Boston, Massachusetts.

"Written especially for private nurses, thus dealing with a phase of work seldom, if ever, the theme of a text-book . . . The hints given are excellent, and we hope they may be widely read."—*British Medical Journal.*

18mo, profusely Illustrated with original Cuts, Tables, &c.; about 260 pp., handsomely bound in leather, 6s.

**A PRACTICAL HAND-BOOK OF MIDWIFERY.** By Francis W. N Haultain, M.D. A practical manual produced in a portable and convenient form for reference, and especially recommended for its compactness, conciseness, and clearness.

"One of the best of its kind, and well fitted to perform the functions its author claims for it."—*Edinburgh Medical Journal.*

Fourth Thousand. Demy 8vo., blue buckram, gilt, with numerous Illustrations, 3s. 6d.

**DIET IN SICKNESS AND IN HEALTH.** By Mrs. Ernest Hart, formerly Student of the Faculty of Medicine of Paris, and of the London School of Medicine for Women. With an Introduction by Sir Henry Thompson, F.R.C.S., M.B.Lond.

"Mrs. Hart speaks not only with the authority derived from experience, but with the ease and freedom of an expert. We have perused this book with great pleasure, and feel sure that many will find in it much to lighten the days of those whose digestion is enfeebled."—*The Hospital.*

New and Revised Edition (10th thousand), with entirely new chapters on Monthly Nursing and Confinements, profusely illustrated with over 100 new Cuts. Crown 8vo., cloth gilt, 3s. 6d.

**NURSING: ITS THEORY AND PRACTICE.** Being a complete Text-Book of Medical, Surgical, and Monthly Nursing. By PERCY G. LEWIS, M.D., M.R.C.S., L.S.A. Always a standard work on Nursing, it now occupies the position of a classic, being used throughout the world in Training Schools and by Private Students.

"We warmly recommend it as a nursing manual . . . full of useful hints and information."—*Birmingham Medical Review.*

Crown 8vo., 200 pp., profusely illustrated with original drawings, list of Instruments required, and a Glossary of Terms, 3s. 6d.

**OPHTHALMIC NURSING.** By Sydney Stephenson, M.B., F.R.C.S.E., Surgeon to the Ophthalmic School, Hanwell, W., &c., &c. A valuable contribution to Nursing literature on a subject hitherto never handled with special reference to Nursing.

"May very advantageously be studied by nurses who are about to enter the Ophthalmic service of a hospital."—*The Lancet.*

In the Press. Crown 8vo., illustrated, cloth, 2s.

**ELEMENTARY PHYSIOLOGY FOR NURSES.** By C. F. Marshall, M.D., B.Sc., F.R.C.S., late Surgical Registrar and Anæsthetist to the Hospital for Sick Children, Great Ormond Street; formerly Platt Physiological Research Scholar in the Owen's College, Manchester.

### "THE BURDETT SERIES" OF POPULAR TEXT-BOOKS ON NURSING.

Handy Pocket Size. Bound in Cloth. Price 1s. each.

No. 1. **PRACTICAL HINTS ON DISTRICT NURSING.** By Amy HUGHES, late Superintendent of the Central Training Home, Queen Victoria's Jubilee Institute for Nurses for the Sick Poor; and of the Metropolitan and National Nursing Association.

"The whole book bristles with good advice and common-sense. It should be in the hands of every district nurse, and be read by all who are interested in the subject."—*British Medical Journal.*

"Exceedingly useful little manual on practical work, district hygiene, advice to nurses, and maternity nursing."—*Newcastle Daily Chronicle.*

No. 2. **THE MATRON'S COURSE.** An Introduction to Hospital and Private Nursing. By Miss S. E. ORME, Lady Superintendent, London Temperance Hospital.

"Short practical lectures . . . readable and instructive. . . . and will prove useful to all who study them."—*Scotsman.*

"May be advantageously read by every woman desirous of taking up hospital nursing."—*Weekly Times and Echo.*

No. 3. **THE MIDWIVES' POCKET BOOK.** By Honnor Morten, Author of "How to Become a Nurse, and How to Succeed"; "The Nurse's Dictionary," &c., &c. [*Shortly.*

No. 4. **FEVER NURSING.** Dr. William Harding. [*June.*

London: THE SCIENTIFIC PRESS, Ltd., 28 & 29, SOUTHAMPTON STREET, STRAND, W.C.

